# Induction of HO-1 by 5, 8-Dihydroxy-4′,7-Dimethoxyflavone via Activation of ROS/p38 MAPK/Nrf2 Attenuates Thrombin-Induced Connective Tissue Growth Factor Expression in Human Cardiac Fibroblasts

**DOI:** 10.1155/2020/1080168

**Published:** 2020-12-03

**Authors:** Chien-Chung Yang, Li-Der Hsiao, Hsin-Hui Lin, Hui-Ching Tseng, Jiro Hasegawa Situmorang, Yann-Lii Leu, Chuen-Mao Yang

**Affiliations:** ^1^Department of Traditional Chinese Medicine, Chang Gung Memorial Hospital at Tao-Yuan, Kwei-San, Tao-Yuan 33302, Taiwan; ^2^School of Traditional Chinese Medicine, College of Medicine, Chang Gung University, Kwei-San, Tao-Yuan 33302, Taiwan; ^3^Department of Pharmacology, College of Medicine, China Medical University, Taichung 40402, Taiwan; ^4^Graduate Institute of Natural Products, College of Medicine, Chang Gung University, Taoyuan 33302, Taiwan; ^5^Ph.D. Program for Biotech Pharmaceutical Industry, China Medical University, Taichung 40402, Taiwan; ^6^Department of Post-Baccalaureate Veterinary Medicine, College of Medical and Health Science, Asia University, Wufeng, Taichung 41354, Taiwan

## Abstract

Heme oxygenase-1 (HO-1) has been shown to exert as an antioxidant and anti-inflammatory enzyme in cardiovascular inflammatory diseases. Flavonoids have been demonstrated to display anti-inflammatory and antioxidant effects through the induction of HO-1. 5,8-Dihydroxy-4′,7-dimethoxyflavone (DDF), one of the flavonoid compounds, is isolated from *Reevesia formosana*. Whether DDF induced HO-1 expression on human cardiac fibroblasts (HCFs) remained unknown. Here, we found that DDF time- and concentration-dependently induced HO-1 protein and mRNA expression, which was attenuated by pretreatment with reactive oxygen species (ROS) scavenger N-acetyl cysteine (NAC) in HCFs. DDF-enhanced ROS generation was attenuated by NAC, but not by either diphenyleneiodonium chloride (DPI, Nox inhibitor) or MitoTempol (mitochondrial ROS scavenger). Interestingly, pretreatment with glutathione (GSH) inhibited DDF-induced HO-1 expression. The ratio of GSH/GSSG was time-dependently decreased in DDF-treated HCFs. DDF-induced HO-1 expression was attenuated by an inhibitor of p38 MAPK (p38i VIII) or siRNA, but not by MEK1/2 (PD98059) or JNK1/2 (SP600125). DDF-stimulated p38 MAPK phosphorylation was inhibited by GSH or p38i VIII. Moreover, DDF-induced HO-1 expression was mediated through Nrf2 phosphorylation and translocation into the nucleus which was attenuated by NAC or p38 siRNA. DDF also stimulated antioxidant response element (ARE) promoter activity which was inhibited by NAC, GSH, or p38i VIII. Interaction between Nrf2 and the ARE-binding sites on the HO-1 promoter was revealed by chromatin immunoprecipitation assay, which was attenuated by NAC, GSH, or p38i VIII. We further evaluated the functional effect of HO-1 expression on the thrombin-induced fibrotic responses. Our result indicated that the induction of HO-1 by DDF can attenuate the thrombin-induced connective tissue growth factor expression. These results suggested that DDF-induced HO-1 expression is, at least, mediated through the activation of the ROS-dependent p38 MAPK/Nrf2 signaling pathway in HCFs. Thus, the upregulation of HO-1 by DDF could be a candidate for the treatment of heart fibrosis.

## 1. Introduction

Growing evidence has indicated that oxidative stress participates in several cardiovascular diseases. In the cardiovascular system, fibroblasts are the most abundant cell population and play an important role in cardiac development, cardiac repair and remodeling, and cardiac fibrosis in response to mechanical force, chemical signals, or various insults. The activation of fibroblasts expresses profibrotic cytokines and positively amplifies the effects via autocrine/paracrine mechanisms. For example, cardiac fibrosis is characterized by excessive deposition of fibrotic extracellular matrix (ECM) and synthesis and deposition of collagen which directly cause left ventricular (LV) dysfunction, alter electrical conduction, and increase coronary resistance [1]. The changes in these functions might be related to the generation of reactive oxygen species (ROS) through the induction of inflammatory mediators via the activation of several signaling components in the cardiac fibroblasts [2, 3]. Accumulating evidence shows that an imbalance between ROS formation and antioxidant defense mechanisms associated with increased inflammatory responses triggers the initiation and progression of cardiovascular diseases. Under pathological conditions, elevated ROS production can result in oxidative damage to DNA, proteins, and lipids and changes in cellular functions leading to cell death. Indeed, several studies have indicated that dysregulated ROS production has a crucial role in a host of cardiac diseases, including heart failure (HF), cardiac hypertrophy, cardiac ischemia-reperfusion injury (I/RI), and ischemic cardiomyopathy [3–6]. Therefore, the development of an effective antioxidant strategy to target these components could provide a beneficial intervention for the management of cardiovascular diseases.

Connective tissue growth factor (CTGF), a cysteine-rich secreted protein, belongs to a set of structurally related proteins of the CCN family. CTGF is involved in angiogenesis and cellular differentiation under normal circumstances. In addition, CTGF also modulates wound healing and fibrosis in pathological conditions [7, 8]. In tissue response to injury and fibrotic reaction, CTGF could stimulate the proliferation of fibroblasts, their differentiation towards myofibroblasts, and enhancement of extracellular matrix (ECM) production. In granulation tissue and various fibrotic disorders, the higher levels of CTGF were detected in various organs including the heart [9]. Therefore, CTGF might play a key role in the development of various cardiac diseases such as myocardial fibrosis [10]. The expression of CTGF, an immediate early gene, is regulated by a variety of cell types and stimuli, including thrombin [11–13]. Altieri et al. found that thrombin triggers primary human atrial fibroblasts to differentiate to myofibroblasts enriched for *α*-smooth muscle actin (*α*SMA), fibronectin, and type I collagen [14]. Dabigatran, a thrombin inhibitor, can attenuate cardiac fibrosis induced by high-pressure overload and improve global cardiac function [14, 15]. Therefore, the expression of CTGF induced by thrombin might be a key player in the pathogenesis of cardiac fibrosis.

Heme oxygenase- (HO-) 1 is considered to be a potential therapeutic target for human diseases, including cardiovascular inflammatory diseases. HO-1 has been shown to protect against inflammatory responses and to be induced by various stimuli and oxidative stresses [16, 17]. HO-1 can suppress cardiomyocyte senescence and improve heart function in myocardial infarction and aged mice [18]. Emerging evidence has revealed that the NF-E2-related factor (Nrf2)/HO-1 signaling pathway is a critical regulator of cardiovascular homeostasis via the suppression of oxidative stress, which can prevent oxidative stress-associated cardiac remodeling and heart failure [19, 20]. Chinese herbal medicines have attracted attention as the treatment of inflammatory diseases. In particular, the Sterculiaceae family has been discovered to exhibit several biological activities, such as anti-inflammatory and antioxidant activities [21, 22]. Several components extracted from Chinese herbal medicines contain flavonoids that possess anti-inflammatory and antioxidant effects on pathophysiological conditions including cardiovascular diseases. 5,8-Dihydroxy-4′,7-dimethoxyflavone (DDF) is one of the flavonoids isolated from *Reevesia formosana*. These studies prompted us to explore whether DDF can attenuate the effects of thrombin on CTGF expression in HCFs.

The upregulation of HO-1 by various stimuli is tightly modulated through different signaling pathways in various types of cells [23]. For instance, EGF-induced HO-1 expression is mediated through EGFR, NADPH oxidase (Nox), and ROS production in HT-29 cells [24]. Mitogen-activated protein kinases (MAPKs) have also been demonstrated to upregulate HO-1 expression in response to diverse stimuli [25, 26]. Sodium arsenite upregulates HO-1 expression via the activation of JNK1/2 in rat hepatocytes [27] and via ERK1/2 and p38 MAPK pathways through the depletion of glutathione (GSH) and the increase in oxidative stress in chicken hepatoma cells [28]. Moreover, there are several transcriptional regulations involved in HO-1 gene expression through the activation of Sp1, activating-protein 1 (AP-1), or Nrf2 modulated by various signaling pathways [29, 30]. Thus, the objective of this study is aimed at dissecting whether DDF can induce HO-1 expression and attenuate the thrombin-induced CTGF expression, shared with similar mechanisms such as Nox/ROS, MAPKs, and Nrf2 in HCFs. The present results revealed that DDF-induced HO-1 expression is, at least, mediated through the activation of the ROS-dependent p38 MAPK/Nrf2 signaling pathway which inhibited the thrombin-induced CTGF expression in HCFs.

## 2. Material and Methods

### 2.1. Materials

**Figure 1:**
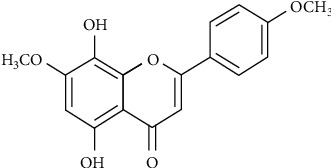
Chemical structure of 5, 8-Dihydroxy-4′,7-dimethoxyflavone (DDF) isolated from *Reevesia formosana*.

Dulbecco's modified Eagle's medium (DMEM)/F-12 and fetal bovine serum (FBS) were purchased from Invitrogen (Carlsbad, CA, USA). 5, 8-Dihydroxy-4′,7-dimethoxyflavone (DDF, Figure 1) was kindly provided by the Natural Products Laboratory of Dr. Yann-Lii Leu (Chang Gung University, Taiwan). Hybond C membrane and enhanced chemiluminescence (ECL) Western blotting detection system were purchased from GE Healthcare Biosciences (Buckinghamshire, England, UK). Antibodies against phospho-p38 and phospho-Nrf2 were purchased from Cell Signaling (Danvers, MA). Actinomycin D (Act.D), cycloheximide (CHI), N-acetyl cysteine, SP600125, GSH, trolox, diphenyleneiodonium chloride (DPI), PD98059, and p38 inhibitor VIII (p38i VIII) were purchased from Biomol (Plymouth Meeting, PA). Anti-*β*-actin antibody was purchased from Biogenesis (Boumemouth, UK). Anti-GAPDH (#MCA-1D4) was obtained from EnCor Biotechnology (Gainesville, FL). Anti-HO-1 pAb and Glutathione (GSSG/GSH) detection kits were purchased from Enzo Life Sciences (Farmingdale, NY). CTGF antibody (sc-25440) was purchased from Santa Cruz (Santa Cruz, CA, USA). Mitotempol, dihydroethidium (DHE), and CM-H_2_DCFDA were purchased from Molecular Probes (Eugene, OR, USA). SDS-PAGE reagents were purchased from MDBio Inc. (Taipei, Taiwan). Thrombin and other chemicals were purchased from Sigma (St. Louis, MO, USA).

### 2.2. Cell Culture and Treatment

Human cardiac fibroblasts (HCFs) were purchased from ScienCell Research Laboratories (San Diego, CA, USA) and cultured in DMEM/F-12 medium supplemented with 10% FBS and antibiotics (100 U/ml penicillin G, 100 ng/ml streptomycin, and 250 ng/ml fungizone) at 37°C in a humidified 5% CO_2_ atmosphere. When the cultures reached confluence, cells were treated with 0.05% trypsin/0.53 mM EDTA for 1 min at 37°C. The cell suspension was diluted with DMEM/F-12 containing 10% FBS to a concentration of 2 × 10^5^ cells/ml. The cell suspension was seeded in (1 ml/well) 12-well culture plates, (2 ml/well) 6-well culture plates, and (10 ml/dish) 10 cm culture dishes and made quiescent at confluence by incubation in serum-free DMEM/F-12 for 24 h. The following experiments were carried out using HCF passages from 5 to 7.

### 2.3. Cell Viability Assay

HCFs were seeded onto 24-well culture plates reaching 70% confluence, serum-starved for 24 h, and then incubated with various concentrations of DDF for 16 h. Cell viability was assessed by Cell Counting Kit-8 (CCK-8) assay through detecting mitochondrial-metabolized formazan generation directly proportional to living HCFs. The absorbance of samples was measured by an ELISA reader (Biotech, H1) with a wavelength of 450 nm.

### 2.4. Preparation of Samples and Western Blot Analysis

HCFs were made quiescent in serum-free DMEM/F-12 for 24 h, incubated without or with different concentrations of DDF at 37°C for the indicated time intervals. When inhibitors were applied, they were added 1 h except indicated before the exposure to DDF. After incubation, the cells were rapidly washed with ice-cold PBS and lysed with a sample buffer containing 125 mM Tris–HCl, 1.25% SDS, 6.25% glycerol, 3.2% *β*-mercaptoethanol, and 7.5 *μ*M bromophenol blue with pH 6.8. Samples were denatured, subjected to SDS-PAGE using a 10% (*w*/*v*) running gel, and transferred to nitrocellulose membrane. The membranes were immunoblotted with one of the primary antibodies (1 : 1000 dilution) overnight at 4°C, followed by incubation with a peroxidase-conjugated secondary antibody at room temperature for 2 h. The immunoreactive bands were visualized by enhanced chemiluminescence reagent (Western Lighting Plus; Perkin Elmer, Waltham, MA, USA). The images of the immunoblots were acquired by using a UVP BioSpectrum 500 imaging system (Upland, CA, USA), and densitometry analysis was conducted using UN-SCAN-IT gel software (Orem, UT, USA).

### 2.5. Total RNA Extraction and Real-Time PCR Analysis

Total RNA was extracted with 1 ml TRIzol (Invitrogen) from HCFs treated with DDF for various time intervals. First strand cDNA synthesis was performed with 2 *μ*g of total RNA using random hexamers as primers in a final volume of 20 *μ*l (5 *μ*g/*μ*l random hexamers, 1 mM dNTPs, 2 units/*μ*l RNase inhibitor, and 10 unit/*μ*l of superscript II reverse transcriptase). The reaction was carried out at 37°C for 60 min. The synthesized cDNAs were used as templates for PCR reaction using Q-Amp™ 2× screening Fire Taq Master mix (Bio-Genesis Technologies, Taipei, Taiwan) and primers for the target genes. qPCR was performed by using Kapa Probe Fast qPCR Kit Master Mix Universal (KAPA Biosystems, Wilmington, MA, USA) on a StepOnePlus™ real-time PCR system (ThermoScientific-Applied Biosystems). The relative amount of the target gene was calculated using the *ΔΔ*Ct method (Ct = threshold cycle). The primer sequences were as follows:

### 2.6. Transient Transfection with siRNAs

HCFs were plated at 2 × 10^5^ cells/ml in 12-well or 10 cm dish until reaching about 70% confluence. Cells were washed once with PBS and then added with 1 ml/well or 5 ml/dish of Opti-MEM medium (Gibco, Grand Island, NY, USA) before transfection. Transient transfection of siRNAs (p38*α* and Nrf2) was carried out using Lipofectamine 2000 transfection reagent (Invitrogen, Carlsbad, CA, USA). The DNA Lipofectamine reagent complex was added to each well to a final concentration of 100 nM siRNA and then incubated at 37°C for 5 h. The cells were shifted to DMEM/F-12 medium containing 10% FBS for an additional 3 h, washed twice with PBS, and then maintained in serum-free DMEM/F-12 for 16 h before treatment with DDF.

### 2.7. Measurement of Intracellular ROS Accumulation

The levels of intracellular ROS were determined using CM-H_2_DCFDA and DHE. The fluorescence intensities of DCF and DHE staining were observed by a fluorescence microscope (Zeiss, Axiovert 200 M). HCFs were washed with warm PBS and incubated in phenol red-free medium containing 10 *μ*M CM-H_2_DCFDA or 5 *μ*M DHE at 37°C for 30 or 10 min, respectively. Subsequently, medium containing DCF-DA and DHE was removed and replaced with fresh medium. HCFs were then incubated with various concentrations of DDF. The fluorescence intensity (relative fluorescence units) was measured at 495 nm excitation and 529 nm emission for DCF or 518 nm excitation and 605 nm emission for DHE using a fluorescence microplate reader (Synergy HT1, BioTek, Winooski, VT, USA).

### 2.8. Glutathione Assay

HCFs were pretreated without or with respective inhibitors for 1 h and then incubated with 10 *μ*M DDF for the indicated time intervals. The supernatants were used to measure the ratio of reduced GSH/oxidized GSH (GSSG) as the marker of oxidative stress, determined using a GSH detection kit, according to the manufacturer's instructions (Enzo Life Sciences, Farmingdale, NY, USA).

### 2.9. Immunofluorescence Staining

HCFs were cultured in 6-well culture plates with coverslips until reaching about 70% confluence and serum-free for 24 h. The cells were treated without or with inhibitor for 1 h and then incubated with DDF for the indicated time intervals. After washing twice with ice-cold PBS, the cells were permeabilized with cold methanol for 10 min, then fixed with 5% BSA in 37°C for 30 min. The staining was followed by incubation with an anti-Nrf2 polyclonal antibody (1 : 100 dilution) with 1% BSA overnight at 4°C, washing thrice with PBS, incubating with fluorescein isothiocyanate- (FITC-) conjugated goat anti-rabbit antibody (1 : 100 dilution) with 1% BSA for 1 h, and washing thrice with PBS. Images were captured with a fluorescence microscope (Axiovert 200 M; Carl Zeiss, Thornwood, NY, USA).

### 2.10. Chromatin Immunoprecipitation (ChIP) Assay

To detect the association between nuclear proteins and human HO-1 promoter, HCFs in the 10 cm dish were grown to confluence and serum-free for 24 h. After treatment with DDF, protein-DNA complexes were fixed by 1% formaldehyde in the medium. The fixed cells were washed and lysed in a SDS-lysis buffer (1% SDS, 5 mM EDTA, 1 mM PMSF, and 50 mM Tris-HCl). The cell lysates were sonicated at 4°C until the DNA size became 200-300 base pairs. The samples were centrifuged, and the soluble chromatin was precleared by incubation with sheared salmon sperm DNA-protein agarose A for 30 min at 4°C with rotation. After precleared, samples were centrifuged and the supernatant was transferred to a new tube. The concentrations of samples were quantified and balanced. One portion of the sample was used as a DNA input control, and the remains were incubated with an anti-phospho-Nrf2 antibody overnight at 4°C. The immunoprecipitating complexes of antibody-protein-DNA were collected by using protein A beads overnight with rotation at 4°C. After incubation, the samples were washed with low-salt buffer (0.1% SDS, 1% Triton A-100, 2 mM EDTA, 20 mM Tris-HCl, pH 8.1, and 150 mM NaCl), high-salt buffer (0.1% SDS, 1% Triton A-100, 2 mM EDTA, 20 mM Tris-HCl, pH 8.1, and 500 mM NaCl), LiCl buffer (0.25 M LiCl, 1% NP-40, 1% deoxycholate, 1 mM EDTA, 10 mM Tris-HCl, and pH 8.1), and Tris-EDTA (pH 8.0), and then eluted with elution buffer (1% SDS, 100 mM NaHCO_3_). The cross-linking of protein-DNA complexes was reversed by incubation at 65°C overnight. The DNA was extracted and resuspended in H_2_O and subjected to PCR amplification using TaqMan ChIP QPCR Assay.

### 2.11. Statistical Analysis of Data

Statistical analysis was performed by using GraphPad Prizm Program 6.0 software (GraphPad, San Diego, CA). We used one-way ANOVA followed by Dunnett's post hoc test when comparing more than two groups of data or nonparametric Kruskal–Wallis test, followed by Dunn's multiple comparison test when comparing multiple independent groups and the ANOVA normality assumptions not met. *P* values of 0.05 were considered to be statistically significant. Post hoc tests were run only if *F* achieved *P* < 0.05 and the homogeneity of variance assumption was met. All the data were expressed as the mean ± SEM, at least three individual experiments (*n* = number of independent cell culture preparations). Error bars were omitted when they fell within the dimensions of the symbols.

## 3. Results

### 3.1. DDF Induced HO-1 Protein and mRNA Expression in HCFs

**Figure 2:**
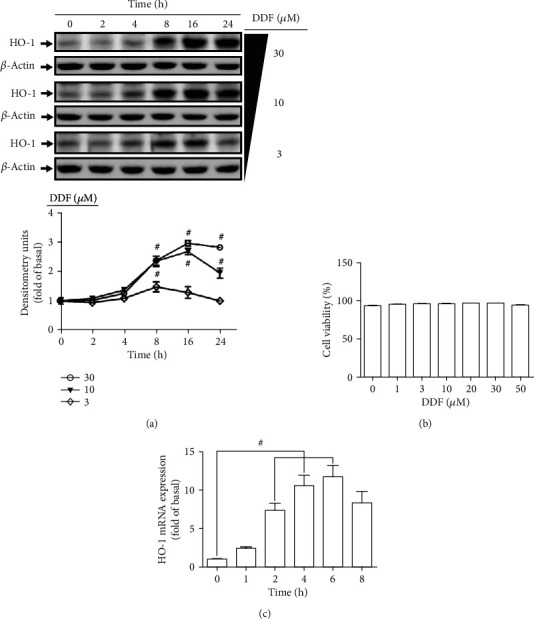
DDF induced HO-1 protein and mRNA expression in HCFs. (a) HCFs were incubated with various concentrations of DDF (3, 10, and 30 *μ*M) for the indicated time intervals (0, 2, 4, 8, 16, and 24 h). The levels of HO-1 and *β*-actin protein expression were examined by Western blot analysis. (b) Cells were incubated with various concentrations of DDF (1, 3, 10, 20, 30, and 50 *μ*M) for 16 h. The cell viability was performed by using a CCK-8 kit. (c) Cells were incubated with 10 *μ*M DDF for the indicated time intervals (0, 1, 2, 4, 6, and 8 h). The levels of HO-1 and GAPDH mRNA were analyzed by real-time PCR. Data are expressed as mean ± SEM of three independent experiments. #*P* < 0.01, as compared with control.

To investigate whether DDF induced HO-1 expression, HCFs were treated with various concentrations (3, 10, and 30 *μ*M) of DDF for the indicated time intervals (0, 2, 4, 8, 16, and 24 h). The levels of HO-1 protein expression were determined by Western blot. As shown in Figure 2(a), DDF induced HO-1 protein expression in a time- and concentration-dependent manner. When cells exposed to 10 and 30 *μ*M within 16 h, there was a maximal increase in HO-1 protein expression by three-fold as compared with that of control. In addition, we found that DDF no greater than 50 *μ*M had no significant effect on cell viability, determined by a CCK8 assay kit (Figure 2(b)). Thus, the concentration of DDF was used at 10 *μ*M for the following experiments.

To further determine the effects of DDF on HO-1 gene transcription, the levels of HO-1 mRNA expression were determined by real-time PCR. HCFs were treated with 10 *μ*M DDF for the indicated time intervals (0, 1, 2, 4, 6, and 8 h). DDF time-dependently induced HO-1 mRNA expression which reached a maximal response within 6 h and slightly declined within 8 h (Figure 2(c)). These results suggested that DDF could induce HO-1 mRNA and protein expression in HCFs.

### 3.2. DDF-Induced HO-1 Expression Requires Ongoing Transcriptional and Translational Processes

**Figure 3:**
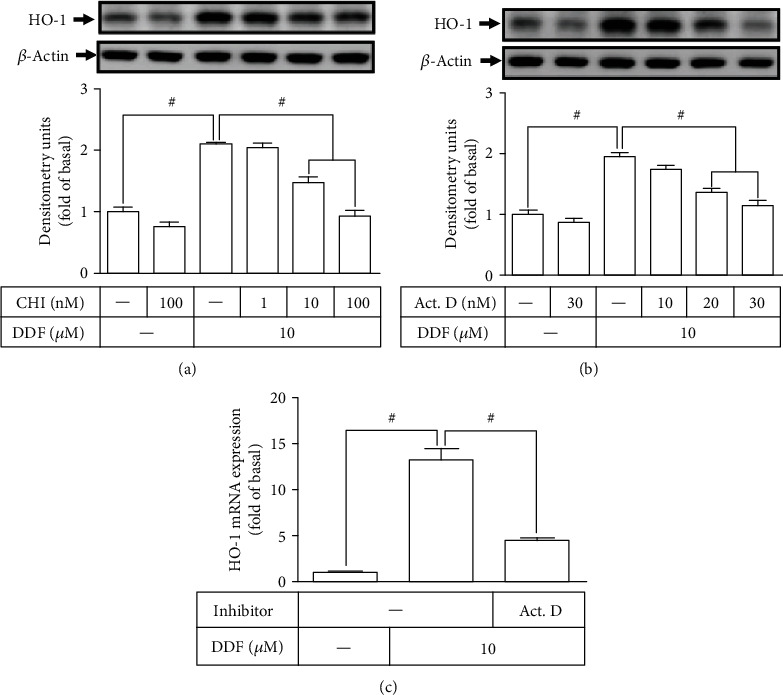
DDF-induced HO-1 expression requires ongoing transcription and translation in HCFs. (a, b) HCFs were pretreated with various concentrations of either (a) CHI or (b) Act. D for 1 h and then incubated with 10 *μ*M DDF for 16 h. The levels of HO-1 and *β*-actin protein expression were examined by Western blot analysis. (c) Cells were pretreated with 30 nM Act. D for 1 h and then incubated with 10 *μ*M DDF for 6 h. The levels of HO-1 and GAPDH mRNA were analyzed by real-time PCR. Data are expressed as mean ± SEM of three independent experiments. #*P* < 0.01, as compared with DDF alone.

To examine whether DDF-induced HO-1 expression mediated through the transcriptional or translational level, HCFs were pretreated with either actinomycin D (Act. D) or cycloheximide (CHI) for 1 h and then incubated with 10 *μ*M DDF for 16 h (protein expression) or 6 h (mRNA expression). Pretreatment with either CHI or Act.D decreased the DDF-induced HO-1 protein expression in a concentration-dependent manner (Figures 3(a) and 3(b)). Further, DDF-induced HO-1 mRNA expression was inhibited by pretreatment with Act. D, analyzed by real-time PCR (Figure 3(c)). These results suggested that DDF-induced HO-1 protein expression is dependent on an increase of HO-1 gene transcription in HCFs.

### 3.3. DDF Induces HO-1 Expression by Stimulation of ROS Generation

**Figure 4:**
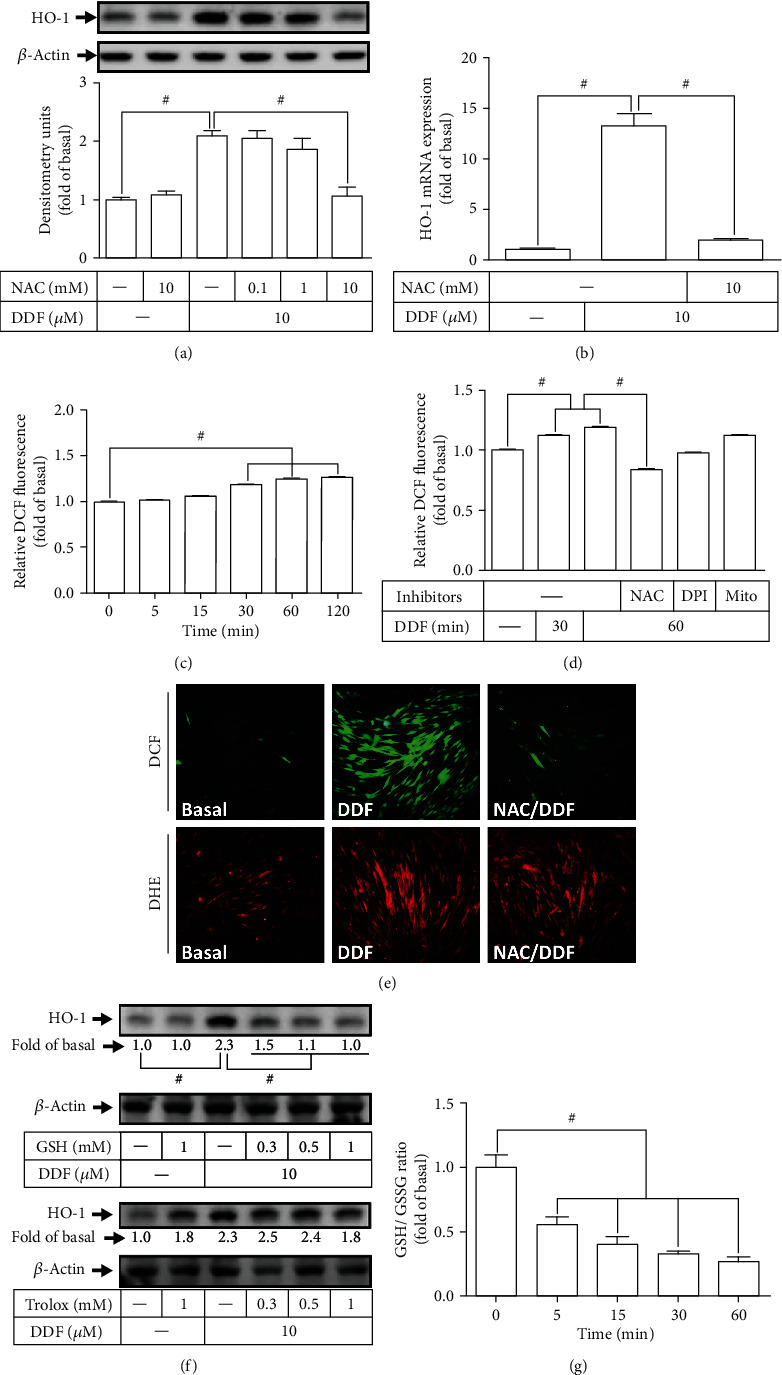
Involvement of ROS generation in DDF-induced HO-1 expression. (a) HCFs were pretreated with various concentrations of NAC for 1 h and then incubated with 10 *μ*M DDF for 16 h. The levels of HO-1 and *β*-actin protein expression were examined by Western blot analysis. (b) Cells were pretreated with 10 mM NAC for 1 h and then incubated with 10 *μ*M DDF for 6 h. The levels of HO-1 and GAPDH mRNA were analyzed by real-time PCR. (c, d) The levels of ROS production were measured. Cells were treated with 10 *μ*M DDF for the indicated time intervals (c). Cells were pretreated without or with 10 mM NAC, 10 *μ*M DPI or 1 *μ*M MitoTempol for 1 h, and then incubated with 10 *μ*M DDF for 30 or 60 min (d). (e) Cells were pretreated with 10 mM NAC for 1 h and then incubated with 10 *μ*M DDF for 1 h, and labeled with DCFDA or DHE. The fluorescence intensities of ROS accumulation were observed using a fluorescence microscope (magnification = 400×). (f) Cells were pretreated with either GSH or Trolox for 1 h and then incubated with 10 *μ*M DDF for 16 h. The levels of HO-1 and *β*-actin protein expression were examined by Western blot analysis. (g) Cells were incubated with 10 *μ*M DDF for the indicated time intervals, and then the ratio of GSG/GSSG was determined by a glutathione detection kit. Data are expressed as mean ± SEM of three independent experiments. #*P* < 0.01, as compared with control.

A previous study has indicated that ROS could act as second messengers in the physiological and pathological responses including induction of HO-1 expression [31, 32]. To determine whether ROS generation is involved in the DDF-induced HO-1 expression, a ROS scavenger of NAC was used for this purpose. As shown in Figures 4(a) and 4(b), pretreatment with NAC concentration dependently attenuated the DDF-induced HO-1 protein and mRNA expression. To directly detect the levels of ROS generation in HCFs treated with 10 *μ*M DDF, the levels of ROS generation were determined by using a fluorescent probe H_2_DCF-DA. We found that DDF enhanced ROS generation in a time-dependent manner with a maximal response (about 1.28 fold) within 1-2 h (Figure 4(c)), which was significantly attenuated by pretreatment with NAC (Figure 4(d)), but not by pretreatment with either DPI (Nox inhibitor) or MitoTempol (mitochondrial ROS scavenger) (Figure 4(d)). These results were further supported by the data of DCF and DHE staining images observed by using a fluorescent microscope (Figure 4(e)). These results demonstrated that induction of HO-1 by DDF is mediated through a ROS-dependent mechanism, but not mediated through either Nox or mitochondrial ROS generation. Further, we investigated whether DDF-induced HO-1 protein expression is mediated through depletion of GSH; GSH was used to test this hypothesis. As shown in Figure 4(f), DDF-induced HO-1 expression was attenuated by pretreatment with GSH in a concentration-dependent manner, but not Trolox. Thus, we speculated that DDF-induced ROS-dependent HO-1 expression may be mediated through the depletion of GSH levels leading to oxidative stress. Further, to ensure that the generation of ROS was due to the imbalance between GSH and GSH disulfide (GSSG), we detected the ratio of GSH/GSSG in HCFs exposed to 10 *μ*M DDF, which was used as a marker of oxidative stress. As shown in Figure 4(g), DDF decreased the ratio of GSH/GSSG which caused ROS accumulation and thereby enhanced HO-1 expression in HCFs.

### 3.4. p38 MAPK Is Required for the Expression of HO-1 Induced by DDF

**Figure 5:**
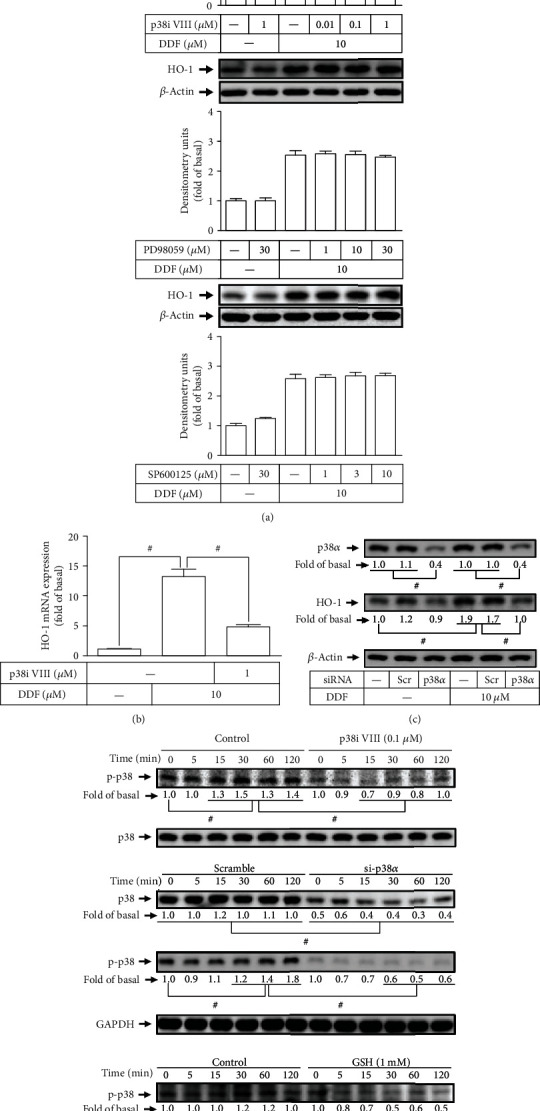
Involvement of p38 MAPK in DDF-induced HO-1 expression but not JNK and Erk. (a) HCFs were pretreated with various concentrations of p38 inhibitor VIII, PD98059, and SP600125 for 1 h, respectively, and then incubated with DDF (10 *μ*M) for 16 h. The protein expression of HO-1 was examined by Western blot analysis. (b) Cells were pretreated with p38 inhibitor VIII for 1 h and then incubated with DDF (10 *μ*M) for 6 h. The levels of HO-1 mRNA were analyzed by real-time PCR. (c) Cells were transfected with scrambled or p38*α* siRNA, and then incubated with DDF (10 *μ*M) 16 h. The protein levels of p38, HO-1, and *β*-actin were determined by Western blot analysis. (d) Cells were treated with DDF (10 *μ*M) for the indicated time intervals with or without preincubation with GSH (1 mM) and p38 inhibitor VIII (0.1 *μ*M), respectively, or transfection with scramble or p38 siRNA. The levels of phospho- and total-p38 MAPK and GAPDH were determined by Western blot. Data are expressed as mean ± SEM of three independent experiments. #*P* < 0.01, as compared with DDF alone.

The activation of MAPK-dependent Nrf2 has been shown to enhance HO-1 expression [26]. To determine whether MAPKs were involved in the DDF induced HO-1 expression, we evaluated the effect of p38 MAPK (p38i VIII), MEK1/2 (PD98059), and JNK1/2 (SP600125) on the DDF-induced HO-1 expression. As shown in Figures 5(a) and 5(b), pretreatment with p38i VIII concentration-dependently decreased HO-1 protein and mRNA expression induced by DDF. However, pretreatment with the inhibitor of MEK1/2 (PD98059) or JNK1/2 (SP600125) failed to change the DDF-induced HO-1 expression (Figure 5(a)). To ensure the role of p38 MAPK in the DDF-induced HO-1 expression, as shown in Figure 5(c), transfection of HCFs with p38 MAPK siRNA knocked down the level of p38 MAPK protein and attenuated the DDF-induced HO-1 protein expression. Furthermore, to investigate whether p38 MAPK phosphorylation was required for HO-1 expression, HCFs were treated with 10 *μ*M DDF for the indicated time intervals (0, 5, 15, 30, 60, and 120 min), and cell extracts were analyzed by Western blot using an antiphosphorylated form of p38 MAPK antibody. As shown in Figure 5(d), DDF stimulated p38 MAPK phosphorylation in a time-dependent manner with a maximal response within 30-120 min. Pretreatment with either GSH or p38i VIII reduced the DDF-stimulated p38 MAPK phosphorylation. Furthermore, to ensure the role of p38 MAPK phosphorylation related to HO-1 expression stimulated by DDF, transfection of HCFs with p38 MAPK siRNA knocked down the level of p38 MAPK protein and attenuated the DDF-stimulated p38 MAPK phosphorylation (Figure 5(d)), but had no effect on HO-1 protein expression during the period of observation (data not shown). These results suggested that DDF-induced HO-1 expression is mediated through activation of ROS/p38 MAPK in HCFs.

### 3.5. DDF Stimulates Nrf2 Nuclear Translocation and Phosphorylation Involved in HO-1 Expression

**Figure 6:**
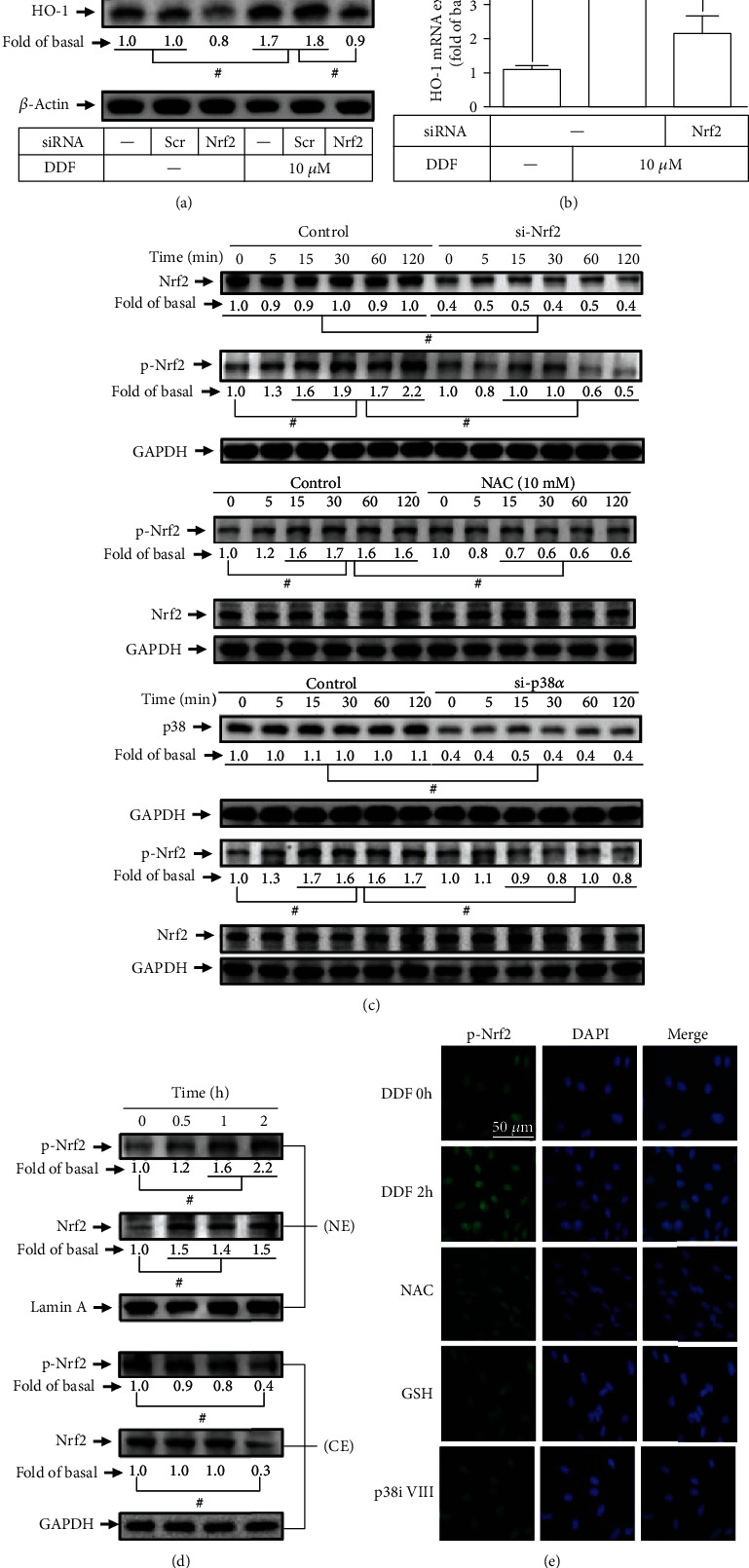
Involvement of Nrf2 in DDF induced HO-1 expression. (a) HCFs were transfected with scrambled or Nrf2 siRNA, and then incubated with DDF (10 U/ml) for 16 h. The protein levels of Nrf2, HO-1, and *β*-actin were determined by Western blot. (b) Cells were transfected with scrambled or Nrf2 siRNA then incubated with DDF for 6 h. The HO-1 mRNA expression was analyzed by real-time PCR. (c) Cells were pretreated with or without NAC (10 mM) or transfected with scrambled siRNA, p38 siRNA, or Nrf2 siRNA, individually, and then incubated with DDF (10 *μ*M) for the indicated time intervals. The levels of phospho- and total-Nrf2, total p38 MAPK, and GAPDH were determined by Western blot. (d) Cells were treated with DDF (10 *μ*M) for the indicated time intervals. Nuclear and cytosolic extracts were prepared, and the protein levels of Nrf2, p-Nrf2, Lamin A, and GAPDH were determined by Western blot. (e) Cells were pretreated with or without NAC (10 mM), GSH (1 mM), or p38i VIII (0.1 *μ*M), then incubated with DDF for 2 h. Phosphorylation of Nrf2 nuclear translocation was labeled with FITC and observed by immunofluorescence microscopy. Scale bars, 50 *μ*m. #*P* < 0.01, as compared with DDF alone.

The activation of Nrf2 has been reported to play an important role in the ARE-driven expression of antioxidant enzymes, including HO-1, which protects against cytotoxicity [33–35]. To investigate whether Nrf2 is involved in the DDF-induced HO-1 expression, transfection of HCFs with Nrf2 siRNA knocked down Nrf2 protein level (Figure 6(a)) and attenuated the DDF-induced HO-1 protein (Figure 6(a)) and mRNA (Figure 6(b)) expression. To determine whether the levels of Nrf2 phosphorylation are related to HO-1 expression, we found that transfection with Nrf2 siRNA knocked down the level of Nrf2 and attenuated the DDF-stimulated Nrf2 phosphorylation (Figure 6(c)). Furthermore, pretreatment with NAC or transfection with p38 siRNA also inhibited Nrf2 phosphorylation stimulated by DDF (Figure 6(c)), suggesting that Nrf2 is a downstream component of ROS/p38 MAPK in HCFs. Activated Nrf2 has been shown to translocate into the nucleus and initiate the expression of antioxidant proteins. Thus, the levels of Nrf2 phosphorylation and translocation into the nucleus were determined by Western blot. We found that DDF time-dependently stimulated Nrf2 phosphorylation and translocation from the cytosol into the nucleus (Figure 6(d)). These results were further supported by the imaging data showing that DDF-stimulated Nrf2 phosphorylation and translocation were observed by immunofluorescent staining coupled to using these pharmacological inhibitors. DDF-induced Nrf2 phosphorylation and translocation into the nucleus were attenuated by pretreatment with NAC, GSH, or p38i VIII (Figure 6(e)).

**Figure 7:**
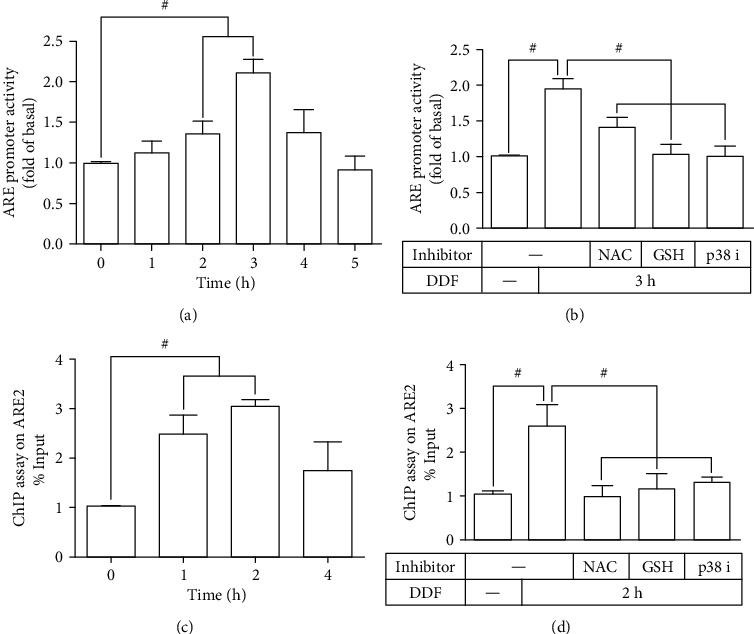
DDF-mediated Nrf2 activates the HO-1 promoter via binding with the ARE site in HCFs. (a, b) HCFs were cotransfected with ARE promoter-Luc and *β*-galactosidase and then incubated with DDF (10 *μ*M) for the indicated time intervals (1, 2, 3, 4, and 5 h) (a); pretreated with or without NAC (10 mM), GSH (1 mM), or p38i VIII (0.1 *μ*M), then incubated with DDF (10 *μ*M) for 3 h (b). The cell lysates were determined in ARE promoter luciferase activity. (c, d) The transcriptional activity of Nrf2 was determined by ChIP assay. Cells were treated with DDF (10 *μ*M) for the indicated time intervals (1, 2, and 4 h) (c). Cells were pretreated with or without NAC (10 mM), GSH (1 mM), or p38i VIII (0.1 *μ*M), then incubated with DDF for 2 h (d). #*P* < 0.01, as compared with DDF alone.

To further examine whether these signaling components and Nrf2 participated in the DDF-regulated ARE transcriptional activity in HCFs, ChIP and luciferase promoter activity assay were performed. As shown in Figure 7(a), DDF time-dependently increased ARE promoter activity with a maximal response within 3 h, which was attenuated by pretreatment with NAC, GSH, or p38i VIII (Figure 7(b)). Moreover, the data of the ChIP assay further revealed that pretreatment with NAC, GSH, or p38i VIII inhibited Nrf2 binding to ARE2 on the HO-1 promoter (Figures 7(c) and 7(d)). Thus, these results demonstrated that DDF-induced HO-1 expression is mediated through the activation of ROS/p38 MAPK leading to Nrf2 binding to the ARE binding site in HCFs.

### 3.6. DDF Attenuates the Thrombin-Induced CTGF Expression

**Figure 8:**
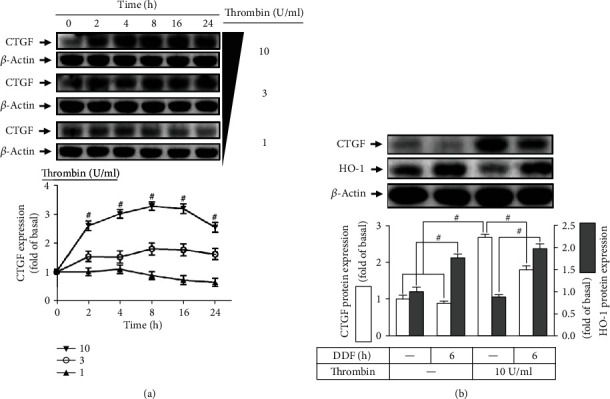
Upregulation HO-1 by DDF inhibits the thrombin-induced CTGF expression. (a) HCFs were incubated with various concentrations of thrombin (1, 3, and 10 U/ml) for the indicated time intervals (0, 2, 4, 8, 16, and 24 h). The levels of CTGF and *β*-actin protein expression were examined by western blot. (b) HCFs were pretreated without or with 10 *μ*M DDF for 6 h and then challenged with 10 U/ml thrombin for 16 h. The levels of CTGF and *β*-actin protein expression were examined by western blot. Data are expressed as mean ± SEM of three independent experiments. #*P* < 0.01, as compared with DDF alone.

To investigate whether DDF exerts an antifibrotic effect reflected as the levels of CTGF expression, HCFs were treated with various concentrations (1, 3, and 10 U/ml) of thrombin for the indicated time intervals (0, 2, 4, 8, 16, and 24 h). The levels of CTGF protein expression were determined by Western blot. As shown in Figure 8(a), thrombin induced CTGF protein expression in a time- and concentration-dependent manner with a maximal expression within 8 h. Pretreatment with DDF for 6 h significantly inhibited the thrombin-induced CTGF expression in HCFs (Figure 8(b)). These results indicated that DDF inhibits the thrombin-induced CTGF expression via, at least in part, the upregulation of HO-1 in HCFs.

## 4. Discussion

**Figure 9:**
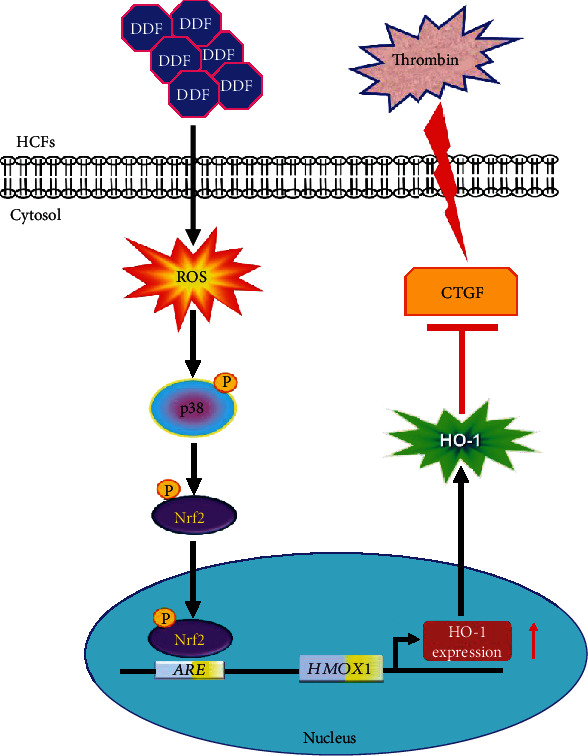
The schematic signaling pathways involved in DDF-induced HO-1 expression inhibited thrombin-stimulated CTGF induction in HCFs. DDF-induced HO-1 expression was, at least in part, mediated through the activation of the ROS-dependent p38 MAPK/Nrf2 pathway, which attenuated the thrombin-stimulated CTGF induction. “⟶” means “activated,” and “⊥” means “inhibited.”

Inflammation and oxidative stress are highly cross-linked processes which play important roles in various cardiovascular inflammatory diseases. HO-1 has been demonstrated to exert as an antioxidant and anti-inflammatory enzyme that ameliorates failing heart-induced hypertrophy, fibrosis, and oxidative stress [36]. Flavonoids are a group of polyphenolic compounds that have been found in fruits, tea, and medical herbs. Several studies have demonstrated that flavonoid compounds display antioxidant and anti-inflammatory effects through the upregulation of antioxidant proteins including HO-1 in various types of cells [26, 37, 38]. However, the mechanisms underlying DDF-induced HO-1 expression were still unknown in HCFs. In this study, we demonstrated that DDF significantly induced HO-1 expression in HCFs. The detailed molecular mechanisms by which DDF induced HO-1 expression were, at least in part, mediated through activation of the ROS-dependent p38 MAPK/Nrf2 pathway and attenuated the thrombin-induced CTGF expression in HCFs (Figure 9). To the best of our knowledge, this is the first report showing that DDF induces *ho-1* gene expression which could prevent and manage cardiovascular inflammatory diseases.

In this study, we found that the *ho-1* gene is activated by DDF in HCFs. DDF time- and concentration-dependently induces HO-1 protein expression, which had no significant effect on cell viability. The upregulation of HO-1 protein by DDF is mediated through a new HO-1 mRNA synthesis, which is attenuated by Act D. Real-time PCR analysis further revealed that DDF induces HO-1 mRNA expression, which is attenuated by Act.D but not cycloheximide in HCFs. These results suggested that DDF-induced *ho-1* gene expression is primarily regulated at the transcriptional level.

Nox family is the major intracellular source of ROS. Nox-dependent ROS generation has been shown to induce HO-1 expression by various stimuli in both *in vitro* and *in vivo* studies [35, 39–42]. In HCFs, we found that DDF could induce ROS generation which was attenuated by pretreatment with NAC (ROS scavenger), but not with DPI (Nox inhibitor) and MitoTempol (mitochondrial ROS scavenger). Further, NAC is able to inhibit the DDF-mediated ROS generation and HO-1 expression. However, the structure of NAC has a thiol (-SH) group which is different from Trolox. Thiol groups are also the major structure of GSH, which act as reducing agents. On the other hand, NAC is also known as a precursor of the synthesis of GSH. The difference between GSH and Trolox on the DDF-induced HO-1 expression may be due to their different chemical properties and reaction with ROS in HCFs. Especially, Trolox has been reported to stimulate Nrf2-mediated HO-1 expression protecting human and murine primary alveolar type II cells from injury by cigarette smoke [43]. In a recent study, flavonoids induced GSH synthesis and HO-1 expression which protected against oxidative stress [44]. In this study, we found that DDF treatment decreased intracellular GSH/GSSG ratio and increased ROS levels leading to HO-1 expression in HCFs. These results demonstrated that DDF-induced HO-1 expression may be due to the depletion of GSH in HCFs, consistent with the report demonstrated by Oguro et al. (1996) [45]. These results were further supported by the data that pretreatment with GSH also attenuated the DDF-induced HO-1 expression in HCFs.

MAPKs consist of three subfamilies, including ERK1/2, JNK1/2, and p38 MAPK which modulate physiological and pathological processes. Several studies have demonstrated that MAPKs relay the signaling from the cell surface into the nucleus [46] involved in the initiation of gene expression such as HO-1 induced by oxidative stress in various types of cells [47–49]. These three MAPKs have been shown to be involved in the expression of HO-1 induced by dihydroquercetin in macrophages and Kupffer cells [26]. The induction of HO-1 by andrographolide in astrocytes [50] and by nitric oxide in HeLa cells [48] partly mediated by p38 MAPK and ERK1/2 signaling. In addition, both JNK1/2 and p38 MAPK have been shown to be involved in lonchocarpine-induced HO-1 expression in brain glial cells [37]. In this study, we found that DDF-induced HO-1 expression was attenuated by the inhibitor of p38 MAPK, but not of MEK1/2 or JNK1/2 in HCFs, suggesting the involvement of p38 MAPK in these responses. These results are consistent with that IL-10 [34], butein [38], and cadmium [49] induced HO-1 expression mediated through p38 MAPK in various types of models. Moreover, we also found that ROS generation can stimulate p38 phosphorylation which was inhibited by pretreatment with NAC and p38 inhibitor, implying that p38 MAPK is a downstream component of ROS-mediated response in HCFs.

The transcription factor Nrf2 is considered to modulate and activate antioxidant response element (ARE) in promoter regions, which regulates the expression of antioxidant and detoxifying genes such as HO-1. The Keap1-Nrf2 pathway is the major regulator of cytoprotective responses to endogenous and exogenous stresses caused by ROS and electrophiles [51]. In normal conditions, Nrf2 is sequestered in the cytoplasm by Keap1 and promotes its degradation by the ubiquitin proteasome. Under the stress conditions, the modification of –SH group on Keap1 or phosphorylation of Nrf2 promotes dissociation of the Nrf2-Keap1 complex. Nrf2 translocates into the nucleus and binds to ARE sequences then increases transcription of Nrf2-regulated phase II antioxidant enzyme, attenuating ROS generation [52]. Oxidative stress refers to elevated intracellular levels of ROS that cause damage to protein, lipid, and DNA. However, recent studies have indicated that a slight increase of ROS is necessary to regulate biological and physiological processes and also benefits cell signaling processes. In HCFs, we found that Nrf2 was involved in the DDF-induced HO-1 expression through the accumulation of phosphorylated Nrf2 in the nucleus of HCFs. Thus, we have observed that pretreatment with NAC, GSH, and p38 inhibitor significantly decreased Nrf2 expression, determined by immunofluorescence staining. Moreover, we further revealed that NAC, GSH, and p38 inhibitor blocked Nrf2 binding to the ARE binding site in the HO-1 promoter. Thus, we suggested that Nrf2 may bind to the ARE sequence in the HO-1 promoter and finally induces HO-1 expression in HCFs.

Thrombin has been shown to play a crucial role in heart hypertrophy and postinjury remodeling processes [14, 53]. Moreover, CTGF is an important component in several pathogeneses of heart diseases [10], which is induced by thrombin [12, 13]. The present results demonstrated that in HCFs, thrombin significantly induced CTGF expression which was attenuated by DDF through the upregulation of HO-1. These findings are consistent with previous studies indicating that inhibition of thrombin response could attenuate cardiac fibrosis and improve cardiac function [14, 15]. Thus, DDF could be beneficial for the treatment of heart failure and cardiac fibrosis. Based on our data, DDF could block CTGF expression induced by thrombin, because pretreatment of DDF with 6 h can inhibit CTGF induction. Huang et al. (2020) found that HO-1 and CTGF present an inverse correlation in a diabetic retinopathy rat model [54]. Riboflavin treatment has beneficial effects on diabetic cardiomyopathy, which could result from raising myocardial HO-1 and decreasing myocardial CTGF levels at the same time [55]. Our data also demonstrated that overexpression of HO-1 can attenuate CTGF expression induced by thrombin in HCFs, this finding verified that the inhibitory effects of DDF on CTGF expression triggered by thrombin, at least partially, come from HO-1 induction. Moreover, the detailed mechanisms by which DDF inhibits thrombin-stimulated CTGF induction is an important issue for further investigation.

## 5. Conclusions

In conclusion, we demonstrated that DDF-induced HO-1 expression is, at least, mediated through the activation of the ROS-dependent p38 MAPK/Nrf2 signaling pathway and attenuates the thrombin-stimulated CTGF induction (Figure 9). Thus, DDF treatment may be a potential therapeutic intervention for the management of heart diseases. However, the limitations of this study were that there was no evidence to clarify the HO-1 expression induced by DDF which protected against the heart diseases *in vivo*. Therefore, it is important to further translate the results of cell culture into an animal study. The results obtained from *in vivo* study could provide the possibility of therapeutic application of DDF in the management of heart diseases.

## Data Availability

The data used to support the findings of this study are available from the corresponding author upon request.

## Abbreviations

Act. D: Actinomycin D
ARE: Antioxidant response element
BSA: Bovine serum albumin
CHI: Cycloheximide
ChIP: Chromatin immunoprecipitation
DCF-DA: 2′, 7′-dichlorofluorescein diacetate
CTGF: Connective tissue growth factor
DHE: Dihydroethidium
DMEM/F-12: Dulbecco's modified Eagle's medium/Ham's F-12
DPI: Diphenyleneiodonium chloride
ECL: Enhanced chemiluminescence
ECM: Extracellular matrix
EGFR: Epidermal growth factor receptor
ERK: Extracellular regulated protein kinase
FBS: Fetal bovine serum
FITC: Fluorescein isothiocyanate
GAPDH: Glyceraldehyde-3-phosphate dehydrogenase
GST: Glutathione S-transferase
HO-1: Heme oxygenase-1
HCF: Human cardiac fibroblast
JNK: c-Jun-NH2-terminal kinase
Keap1: Kelch ECH associating protein 1
LV: Left ventricle
MAPK: Mitogen-activated protein kinase
NAC: *N*-acetyl-L-cysteine
NADPH: Nicotinamide adenine dinucleotide phosphate
Nrf2: NF-E2-related factor 2
PBS: Phosphate-buffered saline
PKC: Protein kinase C
PMSF: Phenylmethylsulfonyl fluoride
ROS: Reactive oxygen species
RT-PCR: Reverse transcription-polymerase chain reaction
SDS-PAGE: Sodium dodecyl sulfate-polyacrylamide gel electrophoresis
SiRNA: Small interfering RNA
Sp1: Specificity protein 1
TNF-*α*: Tumor necrosis factor alpha
Tris-HCl: Tris [hydroxymethyl] aminomethane hydrochloride
TTBS: Tween-Tris-buffered saline
VCAM-1: Vascular cell adhesion molecule-1.
